# Treatment Satisfaction, Efficacy, and Safety of Delgocitinib Ointment Following Switch From Topical Corticosteroids for Trunk and Extremity Rash in Atopic Dermatitis

**DOI:** 10.1111/1346-8138.17915

**Published:** 2025-08-27

**Authors:** Masatoshi Abe, Atsuyuki Igarashi, Hiroyuki Kitajima, Hiroyuki Toyama, Kenji Kabashima, Hidehisa Saeki

**Affiliations:** ^1^ Kojinkai Sapporo Skin Clinic Sapporo Japan; ^2^ Igarashi Dermatology Higashigotanda Tokyo Japan; ^3^ Medical Affairs Department TORII Pharmaceutical Co., Ltd Tokyo Japan; ^4^ Department of Dermatology Kyoto University Graduate School of Medicine Kyoto Japan; ^5^ Department of Dermatology Nippon Medical School Tokyo Japan

**Keywords:** atopic dermatitis, delgocitinib, drug‐related side effects and adverse reactions, extremities, Janus kinase, ointments

## Abstract

Atopic dermatitis (AD) is a chronic inflammatory disease characterized by recurrent remissions and relapses. Topical anti‐inflammatory steroids are commonly used for treatment, but their long‐term use poses concerns because of potential side effects. Delgocitinib ointment, a Janus kinase inhibitor, has demonstrated efficacy in several clinical studies and is expected to be a viable alternative to topical corticosteroids (TCS). To evaluate the real‐world safety and efficacy of delgocitinib in Japanese patients, we assessed the benefits of switching from TCS to delgocitinib ointment in AD patients with rashes on the trunk and extremities. Overall, data from 93 patients (mean age: 35 years) were analyzed. Patients switched from TCS to delgocitinib ointment and were followed for up to 12 weeks. Treatment outcomes were assessed using the treatment Satisfaction questionnaire for medication‐9 (TSQM‐9), Eczema Area and Severity Index (EASI), modified EASI (mEASI), Numerical Rating Scale (NRS) for itching, Atopic Dermatitis Control Tool (ADCT), and Patient Preference Questionnaire (PPQ). During the observation period, TSQM‐9 scores were significantly improved (effectiveness 68.3 to 72.9, *p* < 0.05; global satisfaction 61.7 to 67.9, *p* < 0.01). Additionally, mEASI (8.82 to 6.92, *p* < 0.05), EASI (9.60 to 7.43, *p* < 0.001), NRS (5.6 to 4.5, *p* < 0.001), and ADCT (8.8 to 5.5, *p* < 0.001) scores were decreased during treatment. Moreover, local side effects were improved, with a > 20% reduction in the severity of skin atrophy and telangiectasia. Approximately 72% of patients reported that “the study drug is more effective” using the PPQ. Taken together, our study demonstrates that delgocitinib ointment is an effective treatment option for AD patients with rashes of the trunk and extremities, as well as for those concerned about the potential side effects of TCS.

**Trial Registration:** The study was registered at the Japan Registry of Clinical Trials (jRCTs031230102).

## 1 Introduction

Atopic dermatitis (AD) is a common and chronic inflammatory skin disease, which usually has its onset in early childhood. The main lesion of AD is itchy eczema that fluctuates with remissions and relapses, and this greatly compromises patients' quality of life (QoL). The goal of treatment is to achieve and maintain a state in which symptoms are absent or mild, without disturbance of daily activities, and drug therapy is not required. Even if this level is not fully achieved, the objective is to maintain a mild symptom state without rapid relapses that affect daily activities. Therefore, an essential aspect of AD treatment is inducing remission by rapidly suppressing skin inflammation and pruritus [1, 2].

Topical medications, including anti‐inflammatory drugs such as topical corticosteroids (TCS), as well as ointments containing delgocitinib (a Janus kinase [JAK] inhibitor), tacrolimus (calcineurin inhibitor), and difamilast (a phosphodiesterase 4 inhibitor) are often used as first‐line treatments for acute cases of ad [ 3]. Second treatment options include systemic therapies such as oral cyclosporine and oral JAK inhibitors, subcutaneous injections of biologics, and phototherapy [4]. However, the basic policy of AD treatment remains the use of TCS [1, 2]. The advantages of TCS include their rapid effects, high efficacy, and the ability to select an appropriate strength based on disease severity [3]. The long‐term use of TCS has been associated with local side effects such as skin atrophy and telangiectasia. Additionally, misconceptions about TCS can lead to patient fear and avoidance, resulting in decreased medication adherence. Once rapid remission is achieved with TCS, it is recommended to reduce their use, transition to proactive therapy, or switch to delgocitinib, tacrolimus, or difamilast ointments [1, 2]. Therefore, novel topical treatment options or switching to treatments without TCS that are safe for long‐term use and maintaining patient QoL are required.

Delgocitinib is a small‐molecule JAK inhibitor that has been approved in Japan as the first topical JAK/STAT pathway inhibitor for the treatment of AD. It inhibits all types of the JAK family (JAK1, 2, 3, and tyrosine kinase 2), thereby suppressing immune cells activation [5]. Previous clinical studies have demonstrated the efficacy of delgocitinib ointment in adult patients with ad [ 6, 7, 8, 9, 10]. However, evidence supporting its efficacy is still emerging, and there is limited knowledge regarding its optimal use in combination with or as a replacement for other treatments.

In this study, we evaluated the efficacy of switching to delgocitinib ointment in patients with AD who had been receiving long‐term TCS for more than 3 months and had rashes of the trunk and extremities. Additionally, we examined whether delgocitinib ointment helped reduce local side effects related to TCS treatment.

## 2 Methods

### 2.1 Study Subjects

Patients who visited participating medical institutions between May 2023 and February 2024 and complied with all the following criteria were included in the study. (1) Adults (≥ 18 years old) with AD who provided informed consent. (2) Patients who had been using TCS (excluding the strongest class) on affected areas of the trunk and extremities for at least 3 months. (3) Patients with local side effects, such as skin atrophy and telangiectasia, related to TCS treatment on the trunk and extremities, or those expected to require long‐term TCS treatment with concerns about potential local side effects. (4) Patients whose affected areas on the trunk and extremities could be treated with ≤ 5 g delgocitinib ointment per dose or approximately 30% of the body surface area, adjusted for individual body size. The exclusion criteria were as follows. (1) Patients who had been using the strongest class of TCS. However, the use of clobetasol propionate shampoo on the scalp was permitted. (2) Patients who had been currently using difamilast ointment or delgocitinib ointment. (3) Patients with erosions forming obvious plaques or other lesions in areas where the study drug would be applied. (4) Patients who were pregnant, may be pregnant, or intended to become pregnant during the study period. (5) Patients who were breastfeeding. (6) Patients with prior exposure to delgocitinib ointment. (7) Patients who had received systemic therapy with oral medications (TCS, cyclosporine, or JAK inhibitors) at the time of informed consent or within the past 3 months. (8) Patients who had received systemic therapy with biologics (e.g., dupilumab or nemolizumab) at the time of informed consent or within the past 6 months. (9) Patients who had undergone phototherapy (e.g., ultraviolet therapy) at the time of informed consent or within the past 3 months. (10) Patients who had participated in a clinical study or other studies within the past 6 months. (11) Patients deemed ineligible for the study by investigators or sub‐investigators.

### 2.2 Study Design

From the TCS‐treated rash‐affected areas on the trunk and extremities, one or two areas were selected for switching to the study drug (hereafter referred to as switch areas). The study drug was then applied to these switch areas twice daily (≤ 5 g/time) for 4 weeks at the approved dosage and administration. Switch areas were defined as areas where local side effects such as skin atrophy and telangiectasia related to TCS treatment were observed, or where there was concern about the potential development of such side effects, or where decreased medication adherence to TCS was suspected. After 4 weeks, the condition of the rash in the switch areas was evaluated. If additional rash‐affected areas on the trunk and extremities were identified as suitable for further switching (hereafter referred to as expansion areas), TCS were similarly replaced with the study drug. During the study period, systemic therapy for AD such as oral and injectable medications was prohibited. However, the continuous use of oral antihistamines and herbal medicines indicated for AD was permitted if they have been used at the start of treatment, although any changes in dosage or administration were not allowed.

For any rash in the application area, the initiation or modification of topical medications other than the study drug and moisturizers was prohibited. For any rash outside the application area, the use of the study drug, difamilast ointment, and tacrolimus ointment was not allowed, except that tacrolimus ointment could be used on the head and neck. The use of phototherapy and any interventions that might impact the efficacy evaluation of the study drug was also prohibited.

### 2.3 Target Sample Size

According to a previous survey assessing treatment satisfaction among patients with predominantly mild‐to‐moderate AD using the treatment satisfaction questionnaire for medication‐9 (TSQM‐9), the treatment satisfaction scores for TCS were as follows: 65.3 for effectiveness, 66.1 for convenience, and 65.7 for global satisfaction [6]. Assuming that switching to the study drug would further improve the TSQM‐9 global satisfaction score by 4.5 points, with a standard deviation (SD) of ±15, a power of 0.8, and a significance level of 0.05, approximately 80 patients would be required. Considering potential dropouts, the target enrollment was finally set at 100 patients.

### 2.4 Data Collection and Endpoints

All investigators were provided with standardized training materials for scoring and participated in an orientation session prior to study initiation. The following evaluations were conducted at the start of treatment with the study drug (0 weeks, 0 W), at 1 week, 2, 4, 8, and 12 weeks, or at the time of treatment discontinuation (final evaluation). At enrollment (baseline), patient background information including sex, age, medical history, comorbidities, duration of AD, history of allergic disease, and current therapeutic medications were collected. Local side effects related to TCS treatment (skin atrophy, telangiectasia, and rosacea‐like dermatitis) were assessed using a 5‐point scale: 1 = none (or concern about potential development), 2 = slight (signs are present), 3 = mild, 4 = moderate, and 5 = severe. The TSQM‐9 was used to assess treatment satisfaction. The severity of AD was evaluated using the eczema area and severity index (EASI) for the total area and the modified EASI (mEASI) for the trunk and extremities only. Itching in the application areas was assessed using the numerical rating scale (NRS) for the worst intense at the most itch site and the average level of overall itch intensity. The daily peak of pruritus was evaluated during treatment. Scores were defined from 0, no itch, to 10, the worst itch imaginable. The average of the worst itch imaginable at the most itch site and average level of overall itch intensity was calculated for each time point. The disease control status of AD was evaluated using the atopic dermatitis control tool (ADCT). Patient preference was assessed using the patient preference questionnaire (PPQ) for therapeutic treatment [11], with responses on a 5‐point scale: 0 = strongly disagree, 1 = disagree, 2 = agree, 3 = strongly agree, and 4 = not applicable. The PPQ included the following four questions: (1) “The study drug is more effective than the previous ointment.” (2) “The study drug is easier to use than the previous ointment.” (3) “The study drug has fewer side effects than the previous ointment.” (4) “I prefer the study drug to the previous ointment.” Additionally, patients completed a skin condition questionnaire regarding the study drug application areas, rating the following four aspects on an 11‐point scale: (1) dryness (0 = very dry, 10 = moist), (2) texture (0 = rough, 10 = smooth), (3) firmness and elasticity (0 = not firm or elastic, 10 = firm and elastic), and (4) appearance (0 = poor, 10 = good). For safety endpoints, all adverse events were recorded, and their causal relationships with the study drug and the study itself were evaluated. If worsening of a rash in the study drug application area, an adverse effect related to the study drug (within 0–4 weeks), or the need for acute systemic therapy for AD occurred, these endpoints were recorded, and the study was discontinued for that patient.

The primary endpoint was the change in treatment satisfaction (TSQM‐9) from baseline (0 weeks) to the end of treatment (12 weeks). The secondary endpoints included changes in the EASI, mEASI, itching severity (NRS), disease control (changes in ADCT), severity of local side effects (skin atrophy, telangiectasia, rosacea‐like dermatitis), and skin condition (dryness, texture, firmness, elasticity, appearance) from baseline (0 weeks) to 12 weeks. Patient preference for the study drug (PPQ) at 12 weeks was also evaluated.

### 2.5 Analysis Sets

The primary analysis set was the full analysis set, which included patients who received at least one application of the study drug and had at least one efficacy endpoint assessment. For the safety analysis, the safety analysis set included all enrolled patients, except those who never applied the study drug and those with no available safety data after enrollment.

### 2.6 Statistical Analysis

Statistical analyses were conducted using JMP Ver. 17 (JMP Statistical Discovery LLC, Cary, NC, USA). The analytical approach was as follows: summary statistics, including means ± SD, were calculated to analyze score changes over time. To compare pre‐treatment scores with those at each observation time point, a paired *t*‐test was performed with a two‐sided significance level of 0.05. When analyzing changes over time, missing data—including data from patients who discontinued the study—were generally imputed following the last observation carried forward method. For endpoints requiring a 95% confidence interval (CI), if missing data after the study start were necessary for accurate CI calculation, no imputation was performed; patients with missing data were excluded from the analysis. No imputation was applied to other missing values.

## 3 Results

### 3.1 Backgrounds of Study Subjects

Overall, 101 patients (44 males and 57 females) with a mean age of 35.1 years (34.2 years for males and 35.8 years for females) who provided written informed consent were enrolled in the study. Of these, 93 patients were included in the analysis after excluding 1 patient who withdrew informed consent, 1 patient who did not attend the hospital after enrollment, 4 patients who violated the eligibility criteria related to the use of prohibited concomitant medications, and 2 patients who met the exclusion criteria. At the time of baseline, the overall mean age of the study participants was 34.9 ± 12.5 years, and the mean duration of AD was 21.4 ± 13.8 years. There were no significant differences between the sexes in these baseline characteristics. The overall mean EASI score was 9.6 ± 8.8, and the mean mEASI score for the trunk and extremities was 8.8 ± 8.2. The evaluation of itching in the trunk and extremities using the NRS showed that the average of the itch intensity at the most itch site was 5.6 ± 2.4, and the average level of overall itch intensity was 3.4 ± 1.9. The most common comorbid allergic condition was allergic rhinitis, present in 21 patients, followed by asthma in 2 patients, urticaria in 2 patients, food allergy in 1 patient, and allergic conjunctivitis in 1 patient (Table 1).

**TABLE 1:** Patient backgrounds.

	Total *n* = 93	Male *n* = 41	Female *n* = 52
Age, years (mean ± SD)	34.9 ± 12.5	33.7 ± 12.3	35.8 ± 12.8
(min–max)	(18–79)	(18–79)	(18–63)
Duration of AD^a^ (mean ± SD)	21.4 ± 13.8	22.2 ± 14.1	20.7 ± 13.6
EASI (mean ± SD)	9.6 ± 8.8	12.2 ± 9.9	7.5 ± 7.3
mEASI (trunk and extremities) (mean ± SD)	8.8 ± 8.2	11.1 ± 9.3	7.0 ± 6.8
NRS (worst intense itching) (mean ± SD)	5.6 ± 2.4	5.8 ± 2.7	5.5 ± 2.1
NRS (average itching) (mean ± SD)	3.4 ± 1.9	3.5 ± 2.1	3.3 ± 1.7
Allergic disease (*n*)			
Allergic rhinitis	21	7	14
Asthma	2	0	2
Urticaria	2	0	2
Food allergy	1	1	0
Allergic conjunctivitis	1	1	0
Medication history			
Moisturizer	87 (93.5%)	38	49
Oral antihistamines	57 (61.3%)	29	28

Abbreviations: AD, atopic dermatitis; EASI, Eczema Area and Severity Index; mEASI, modified EASI; NRS, Numerical Rating Scale; SD, standard deviation.

^a^ Unknown duration of AD: 28 patients (17 women and 11 men).

Table 2 shows the history of local anti‐inflammatory drug use on the trunk and extremities at the time of baseline as follows. For the trunk, medium TCS were used by 10 patients (10.8%), strong TCS were used by 29 patients (32.2%), very strong TCS were used by 50 patients (53.8%), and TCS were not used by 11 patients (11.8%). For the upper extremities, medium strength TCS were used by 8 patients (8.6%), strong TCS were used by 35 patients (37.6%), very strong TCS were used by 54 patients (58.1%), and TCS were not used by 5 patients (5.4%). For the lower extremities, medium TCS were used by 6 patients (6.5%), strong TCS were used by 23 patients (24.7%), very strong TCS were used by 45 patients (48.4%), and TCS were not used by 22 patients (23.7%). Regarding tacrolimus ointment, the 0.1% formulation was used on the upper extremities by 1 patient (1.1%). Overall, 87 patients (93.5%) used moisturizer on their trunk and extremities, and 57 patients (61.3%) were taking oral antihistamines.

**TABLE 2:** The history of topical anti‐inflammatory drugs on the trunk and extremities (at the time of enrollment).

	Trunk (*n* = 93)	Upper extremities (*n* = 93)	Lower extremities (*n* = 93)
None	11 (11.8%)	5 (5.4%)	22 (23.7%)
TCS strength			
Weak	0 (0%)	0 (0%)	0 (0%)
Medium	10 (10.8%)	8 (8.6%)	6 (6.5%)
Strong	29 (31.2%)	35 (37.6%)	23 (24.7%)
Very strong	50 (53.8%)	54 (58.1%)	45 (48.4%)
Tacrolimus ointment 0.1%	0 (0%)	1 (1.1%)	0 (0%)

Abbreviation: TCS, topical corticosteroids.

Overall, there were 121 switch areas from 93 patients who were changed to delgocitinib ointment at 0 weeks. The distribution of switched TCS was as follows: overall, medium strength TCS in 12 patients (9.9%), strong TCS in 41 patients (33.9%), and very strong TCS in 68 patients (56.2%). By region, on the trunk, medium TCS were used in 3 patients (13.6%), strong TCS in 9 patients (40.9%), and very strong TCS in 10 patients (45.5%). On the upper extremities, medium TCS were used in 9 patients (9.6%), strong TCS in 32 patients (34.0%), and very strong TCS in 53 patients (56.4%). On the lower extremities, very strong TCS were used in five patients (100%) (Table 3).

**TABLE 3:** Classification of TCS strengths used on switch areas at the time of enrollment.

	*n*	TCS strength			
		Weak	Medium	Strong	Very strong
Trunk	22	0 (0%)	3 (13.6%)	9 (40.9%)	10 (45.5%)
Upper extremities	94	0 (0%)	9 (9.6%)	32 (34.0%)	53 (56.4%)
Lower extremities	5	0 (0%)	0 (0%)	0 (0%)	5 (100%)
Total	121	0 (0%)	12 (9.9%)	41 (33.9%)	68 (56.2%)

*Note: n* = 93 patients, 121 areas.

Abbreviation: TCS, topical corticosteroids.

The frequency of local side effects in the 121 areas from 93 patients who were switched to delgocitinib ointment is shown in Table 4. Skin atrophy was observed in a total 77.7% of the switch areas; 7.4% on the trunk, 66.1% on the upper extremities, and 4.1% on the lower extremities. Telangiectasia was observed in a total 52.9% of the switch areas; 2.5% on the trunk, 48.8% on the upper extremities, and 1.7% on the lower extremities. The frequencies of these side effects were highest in the upper extremities.

**TABLE 4:** Local side effects observed in switch areas at the time of enrollment listed by region.

	Trunk		Upper extremities		Lower extremities		Total	
	*n*	%	*n*	%	*n*	%	*n*	%
Skin atrophy								
Slight	4	3.3	39	32.2	0	0	43	35.5
Mild	5	4.1	32	26.4	5	4.1	42	34.7
Moderate	0	0	9	7.4	0	0	9	7.4
Severe	0	0	0	0	0	0	0	0
Total	9	7.4	80	66.1	5	4.1	94	77.7
Telangiectasia								
Slight	2	1.7	34	28.1	0	0	36	29.8
Mild	1	0.8	16	13.2	2	1.7	19	15.7
Moderate	0	0	8	6.6	0	0	8	6.6
Severe	0	0	1	0.8	0	0	1	0.8
Total	3	2.5	59	48.8	2	1.7	64	52.9

*Note: n* = 93 patients, 121 areas.

### 3.2 Primary Endpoint

**FIGURE 1:**
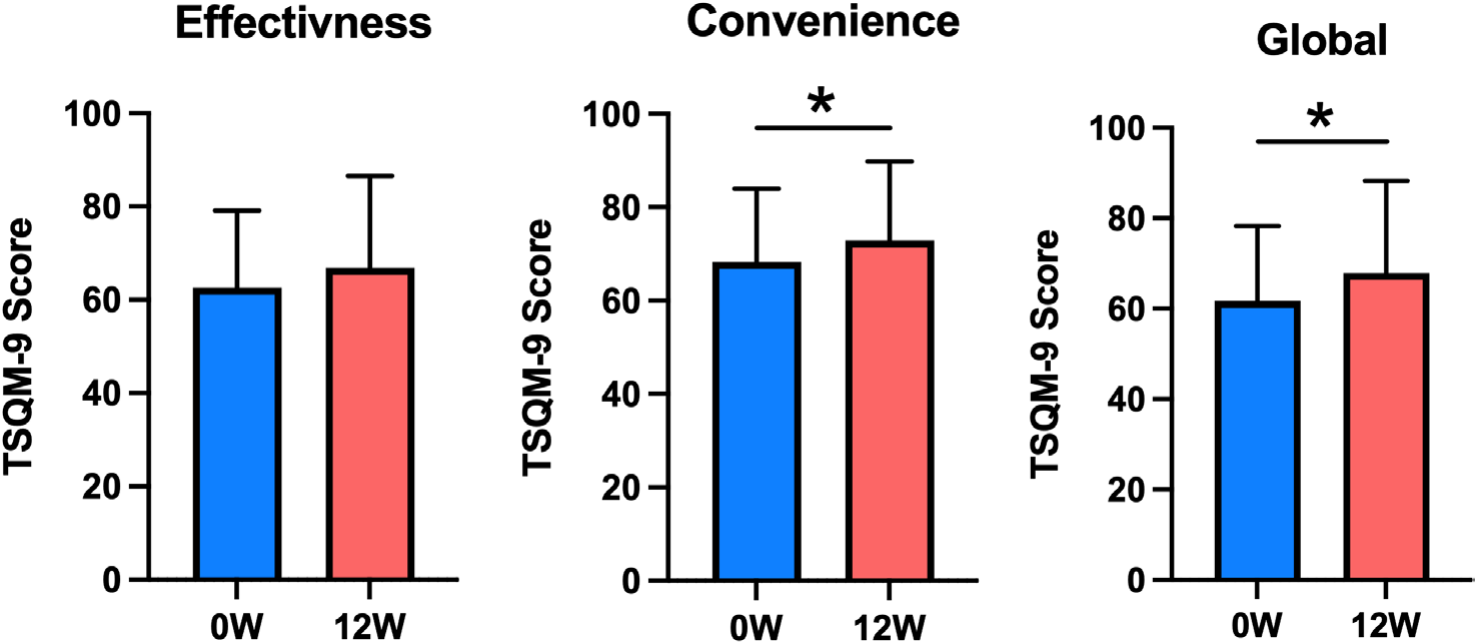
Trends in the treatment satisfaction questionnaire for medication‐9 scores for effectiveness, convenience, and global satisfaction related to rashes on the trunk and extremities in patients with atopic dermatitis at 0 weeks (0 W, blue) and 12 weeks (12 W, red). *n* = 93 in each group. Values are the mean ± standard deviation. *p < 0.05 (paired *t*‐test).

Changes in the TSQM‐9 scores from 0 (at baseline) to 12 weeks are shown in Figure 1. When comparing the scores at 12 weeks with those at 0 weeks, a significant increase in convenience was observed, from 68.3 to 72.9 (*p* < 0.05). An increase in effectiveness was also noted, although the difference was not statistically significant (62.6 to 66.9, *p* = 0.064). Additionally, a significant increase in global satisfaction was observed (61.7 to 67.9, *p* < 0.01).

### 3.3 Secondary Endpoints

**FIGURE 2:**
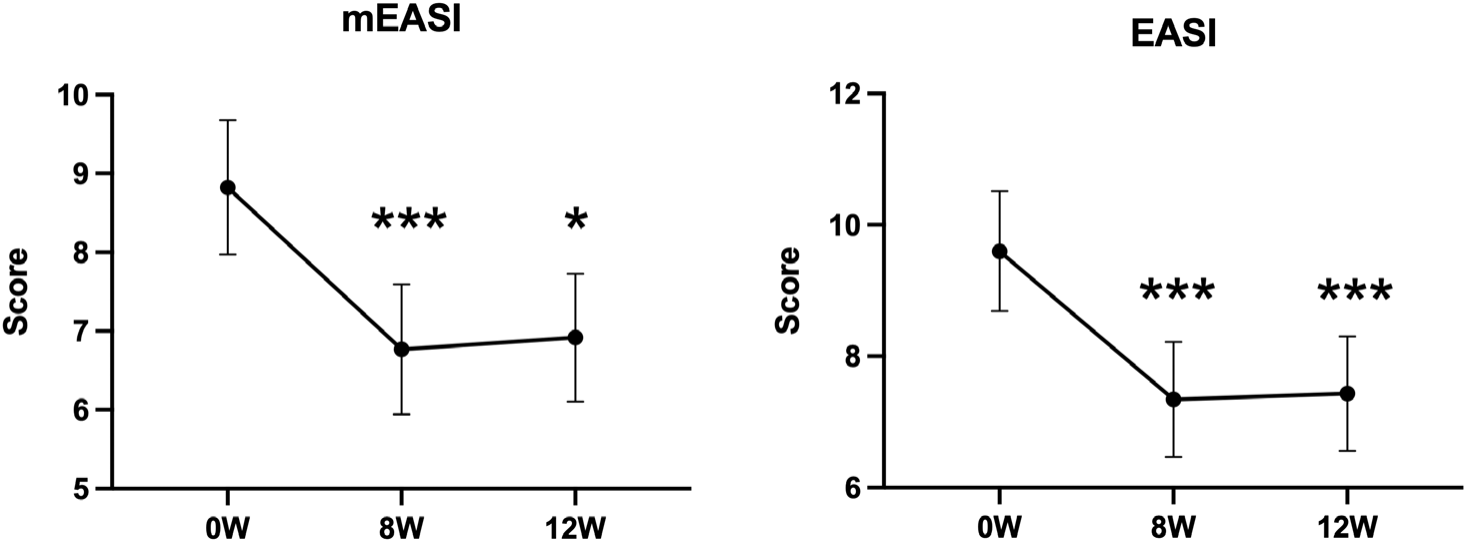
Changes in the eczema area and severity index (EASI) score from 0 to 12 weeks (W) after delgocitinib treatment. Modified EASI (mEASI) scores for the trunk and extremities only are shown on the left, and EASI scores for the total area are shown on the right. *n* = 93 in each group. Values are the mean 95% confidence interval. *p < 0.05; ***p < 0.001 (paired *t*‐test). W, weeks.

**FIGURE 3:**
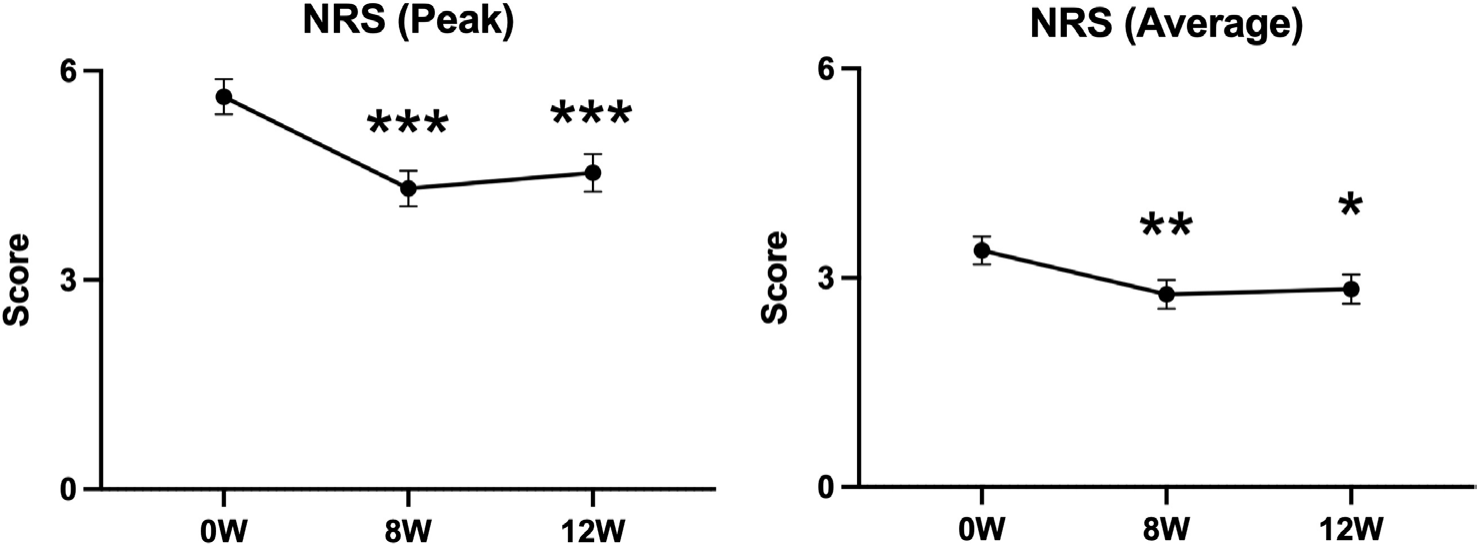
Changes in numerical rating scale (NRS) symptoms on trunk and extremities over time. The daily peak pruritus was evaluated at 0 (*n* = 89), 8 (*n* = 93), and 12 weeks (*n* = 93). Scores were defined as follows: 0; no itch to 10; the worst itch imaginable. The average level of itch intensity at the most itch site is shown on the left (peak), while the average level of overall itch intensity is shown on the right (average). Values are the mean 95% confidence interval. *p < 0.05, **p < 0.01, ***p < 0.001 (paired *t*‐test). At registration (0 weeks), 4 out of 93 patients could not complete the NRS assessment. W, weeks.

**FIGURE 4:**
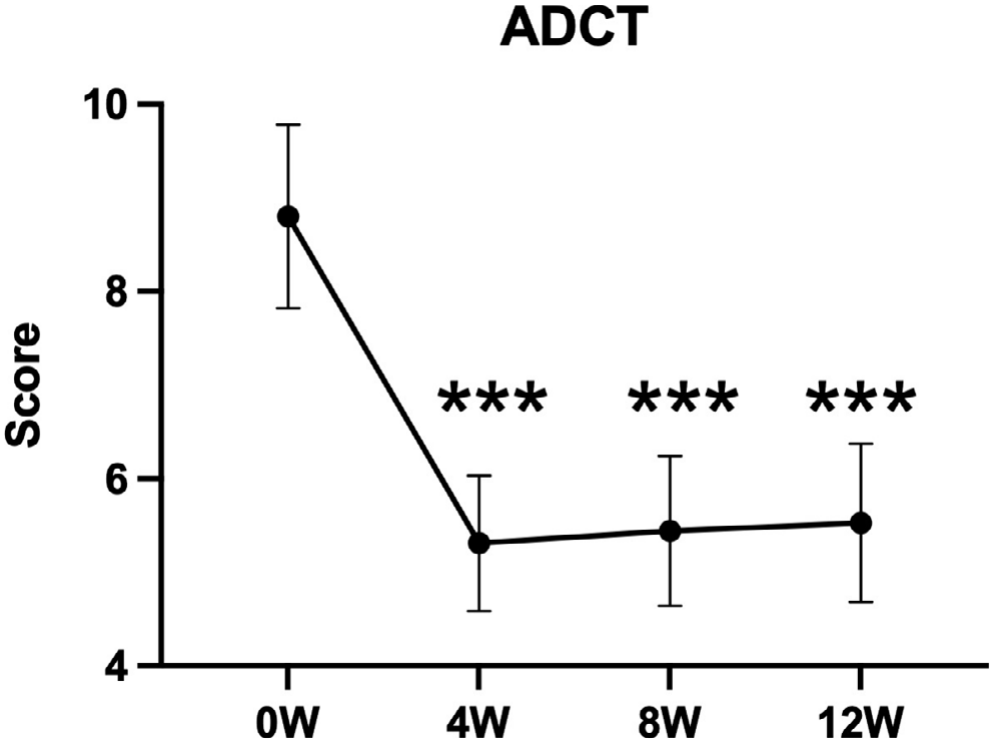
Changes in the atopic dermatitis control tool (ADCT) scores during treatment. ADCT was evaluated at 0 (*n* = 91), 4, 8, and 12 weeks (*n* = 93 respectively). Values are the mean 95% confidence interval. ***p < 0.001 (paired *t*‐test). At registration (0 weeks), 2 out of 93 patients could not complete the ADCT assessment. W, weeks.

**FIGURE 5:**
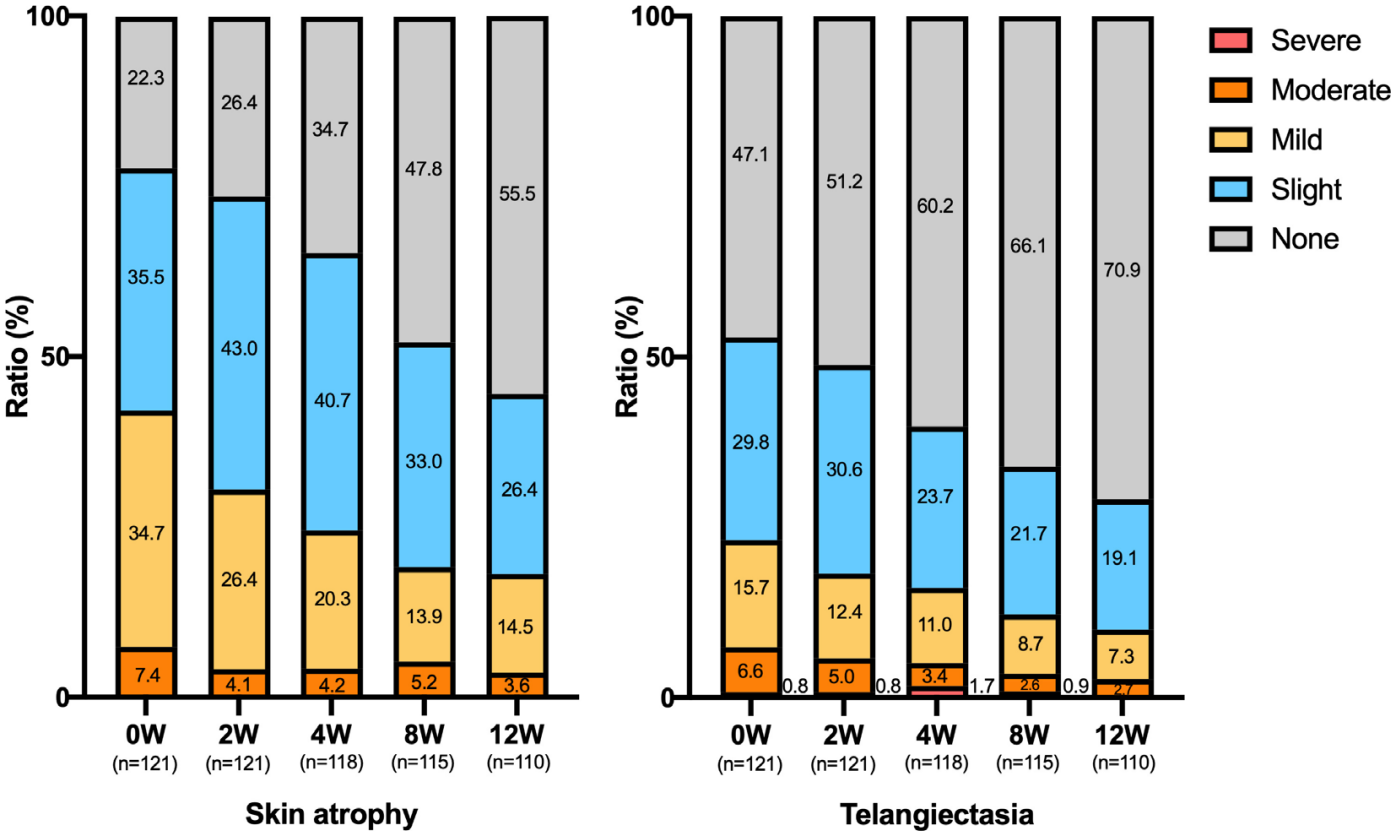
Trends in the severity of local side effects in switch areas. Local side effects related to TCS treatment (skin atrophy, telangiectasia, and rosacea‐like dermatitis) were assessed using a 5‐point scale: 1 = none (or concern about potential development), 2 = slight (signs are observed), 3 = mild, 4 = moderate, and 5 = severe. Percentages of severity rank at 0 (*n* = 121), 2 (*n* = 121), 4 (*n* = 118), 8 (*n* = 115), and 12 weeks (*n* = 110) related to skin atrophy and telangiectasia are indicated. The number of patients decreased at 4–12 weeks because of the early completion of treatment or lack of hospital visits. TCS; topical corticosteroids, W, weeks.

**FIGURE 6:**
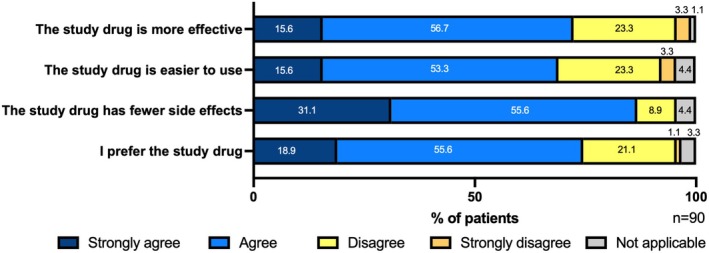
Evaluation of patient preference for therapeutic treatment (PPQ). Patient preference was assessed using the PPQ for therapeutic treatment, with responses assessed on a 5‐point scale: 0 = strongly disagree, 1 = disagree, 2 = agree, 3 = strongly agree, and 4 = not applicable. The PPQ included the following four questions: (1) “The study drug is more effective than the previous ointment.” (2) “The study drug is easier to use than the previous ointment.” (3) “The study drug has fewer side effects than the previous ointment.” and (4) “I prefer the study drug to the previous ointment.” *n* = 90. Imputation was performed using the last observation carried forward method for patients whose condition worsened or who discontinued treatment.

**FIGURE 7:**
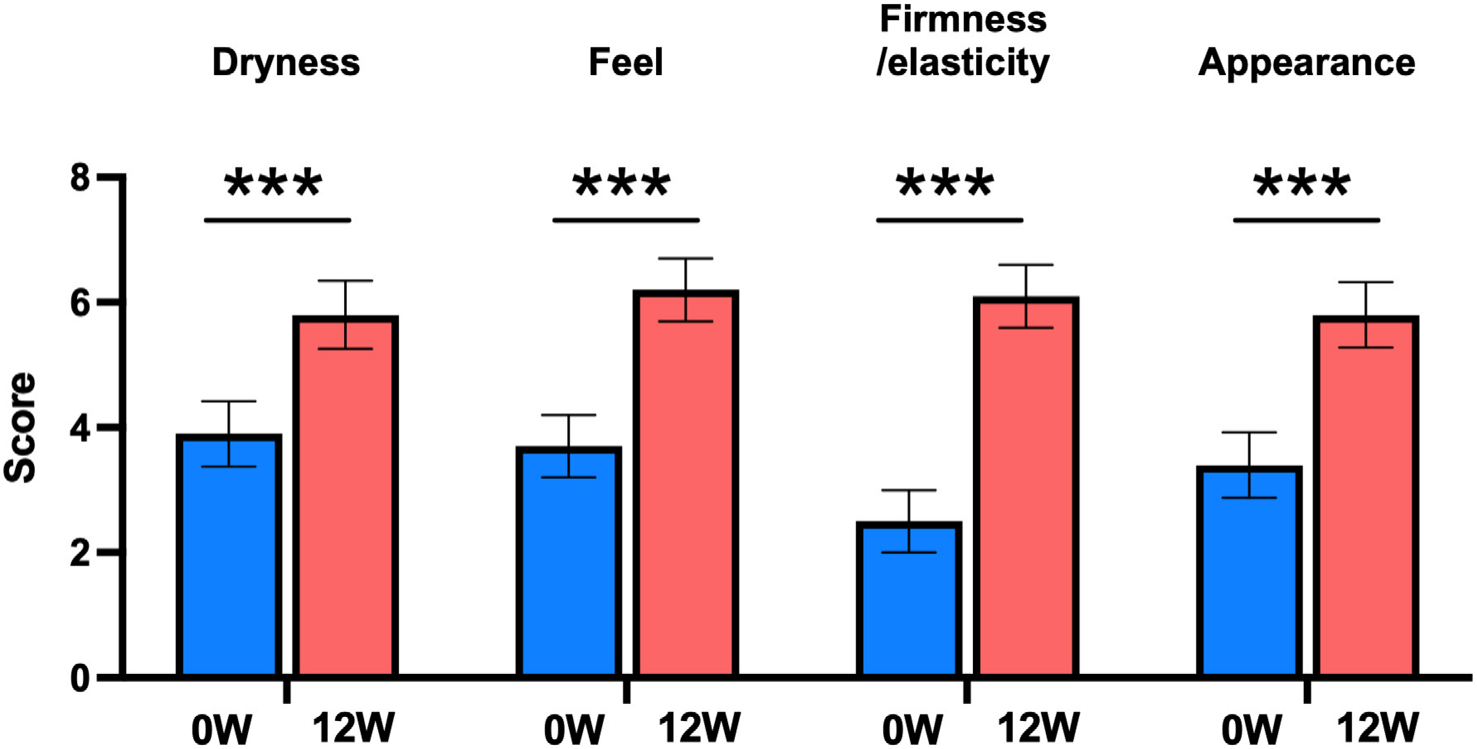
Evaluation of skin condition after treatment. Patients completed a skin condition questionnaire regarding the study drug application areas, rating the following four aspects on an 11‐point scale: (1) dryness (0 = very dry, 10 = moist), (2) texture (0 = rough, 10 = smooth), (3) firmness and elasticity (0 = not firm or elastic, 10 = firm and elastic), and (4) appearance (0 = poor, 10 = good) at 0 (blue, *n* = 91) and 12 weeks (red, *n* = 90). Values are the mean 95% confidence interval. ***p < 0.001 (Wilcoxon signed‐rank test). W, weeks.

There was a significant decrease in the change in mEASI and EASI scores for the trunk and extremities over time (mEASI [8.82 to 6.77 or 6.92, *p* < 0.05], EASI [9.60 to 7.40 or 7.43, *p* < 0.001]) at 8 and 12 weeks compared with 0 weeks (Figure 2). Similarly, significant decreases in the NRS score for the worst imaginable itch intensity at the most itch site (peak) and average level of overall itch intensity (average) were observed (peak 5.6 to 4.3 or 4.5, *p* < 0.001; average 3.4 to 2.7 or 2.8, *p* < 0.05) at 8 and 12 weeks after the treatment (Figure 3). Regarding disease control status assessed by the ADCT, a significant improvement was noted starting at 4 weeks, with good disease control maintained through to 8 to 12 weeks (8.8 to 5.3, 5.4 or 5.5, *p* < 0.001), during the treatment period (Figure 4). Local side effects related to the use of TCS in the switch areas at 0 weeks, including changes in skin atrophy and telangiectasia, over time are shown in Figure 5. The proportion of mild‐to‐severe skin atrophy and telangiectasia was decreased compared with that at 0 weeks. Similarly, in the expansion areas, the proportions of mild‐to‐severe skin atrophy and telangiectasia were also reduced (data not shown). The evaluation of preference (PPQ) comparing the study drug with former treatments demonstrated the proportion of patients who answered “strongly agree” or “agree” was as follows: 72.3% to the question “The study drug is more effective,” 68.9% to “The study drug is easier to use,” 86.7% to “The study drug has fewer side effects,” and 74.5% to “I prefer the study drug” (Figure 6). Additionally, the evaluation of skin condition by the study participants demonstrated the scores for all items including dryness, texture, firmness, elasticity, and appearance were significantly higher at 12 weeks compared with those at 0 weeks (Figure 7). These results indicated that itching improved after switching to delgocitinib ointment, and the subjects tended to be more satisfied with its use.

### 3.4 Treatment Interruption

Six patients discontinued the study because of worsening rashes at the application sites of the study drug or in other areas. Specifically, two patients withdrew because of rash exacerbation at the application sites, one patient because of worsening rashes in other areas, and three patients because of rashes worsening at the application sites and in other areas. All six patients who discontinued the study because of exacerbations had previously been treated with very strong TCS (Table 5).

**TABLE 5:** Data of patients who discontinued the study because of rash exacerbation.

No.	Exacerbated area	Timing	Sex	Age (years)	Duration of AD (years)	Previous treatment on the application areas of the study drug				EASI scores		Clinical history	Comorbidities
						At the time of switch at 0 W		At the time of expansion at 4 W		0 W	At the time of discontinuation		
						Region	Rank of TCS	Region	Rank of TCS				
1	Application areas of the study drug	4 weeks after the start of treatment	Male	39	33	Upper extremities	Very strong	—	—	3.0	4.4	—	Tinea and acne vulgaris
2	Application areas of the study drug	8 weeks after the start of treatment	Female	52	39	Upper extremities	Very strong	No expansion	—	21.0	10	Drug eruption	Allergic rhinitis, panic disorder, and menopausal disorders
3	Other areas	4 weeks after the start of treatment	Female	22	22	Upper extremities	Very strong	—	—	9.1	24.4	—	Post‐inflammatory hyperpigmentation and acne vulgaris
4	Application areas of the study drug and other areas	1–2 weeks after the start of treatment	Male	19	18	Upper extremities	Very strong	—	—	16.5	27.7	—	—
5	Application areas of the study drug and other areas	8 weeks after the start of treatment	Female	27	3	Upper extremities	Very strong	Trunk and lower extremities	Very strong	10.0	16.8	Herpes labialis	—
6	Application areas of the study drug and other areas	4 weeks after the start of treatment	Male	44	44	Upper extremities	Very strong	—	—	38.4	52.0	Herpes zoster and cellulitis	—

Abbreviations: AD, atopic dermatitis; EASI, Eczema Area and Severity Index; TCS, topical corticosteroids; W, weeks.

### 3.5 Safety Evaluation

One patient experienced an adverse event (folliculitis) attributed to this clinical study. This event did not impact the patient's participation in the study; it gradually became less severe.

## 4 Discussion

This study evaluated the efficacy and safety of switching to delgocitinib ointment in patients who had been using TCS for a rash on the trunk and extremities for more than 3 months. The results indicated that switching to delgocitinib ointment helped improve patient satisfaction with the treatment (TSQM‐9), alleviated rash and itching (EASI, mEASI, NRS, and ADCT), and reduced local side effects associated with TCS, such as skin atrophy and telangiectasia. These findings suggest that delgocitinib ointment is likely to be an alternative therapy, offering a potential reduction in the local side effects related to TCS treatment, as well as effective treatment for affected areas, and improved patient satisfaction.

Delgocitinib is a novel pan‐JAK inhibitor that ameliorates the major symptoms of AD, including skin inflammation, pruritus, and impaired skin barrier function [3, 5]. In Japan, two concentrations (0.25% and 0.5%) of delgocitinib ointment are available for ad [ 12]. In the clinical studies involving Japanese patients with mild‐to‐severe AD, 0.5% delgocitinib ointment significantly improved skin rash scores, demonstrating efficacy and safety in the acute phase as well as over the long term (52 weeks) [7, 8, 9, 10]. AD affects approximately 20% of infants, 6% of school‐aged children, and 5% of adults worldwide, making it the most burdensome skin condition globally [13]. Two meta‐analyses of 149 clinical studies involving more than 28 000 children and adults with moderate‐to‐severe AD, as well as 291 trials including 45 846 patients, have suggested that delgocitinib ointment may be as effective as TCS while having a lower risk for local side effects [14, 15]. A recent study reported the efficacy of increasing the delgocitinib ointment concentration from 0.25% to 0.5% in children with AD who were followed up for 56 weeks. The study also found that delgocitinib was better tolerated in pediatric trials compared with TCS and/or tacrolimus ointment [16]. In the present study, the global satisfaction scores (TSQM‐9) were significantly increased at 12 weeks compared with those at 0 weeks, indicating high patient satisfaction with the treatment. “Convenience” was also significantly increased, suggesting that patients appreciated the ease of application and minimal impact on their daily life. Although “effectiveness” tended to increase, the difference was not statistically significant, confirming that patients were satisfied with the delgocitinib treatment, which was not inferior to TCS. In terms of rash improvement, a significant reduction in mEASI scores for the trunk and extremities, as well as in itching severity (NRS), was observed at 8 and 12 weeks compared with that at 0 weeks. Furthermore, in the assessment of disease control status (ADCT), a significant decrease was observed as early as 4 weeks, with good disease control maintained through to the end of the 12‐week observation period. Consistent with previous studies, our data support the efficacy and patient satisfaction of delgocitinib treatment.

TCS have potent anti‐inflammatory effects and are highly effective at managing acute‐phase inflammation; however, their long‐term use increases the risk of local side effects [1, 2]. In addition to these local side effects, the general apprehension about TCS use can reduce medication adherence and make patients hesitant to receive treatment [17, 18]. Indeed, a previous study reported that 80.7% of patients with AD have a fear of TCS use, and 36% exhibit non‐medical adherence to TCS treatment [19]. It is well known that many local side effects caused by TCS can improve upon discontinuation of the drug or with appropriate treatment [20]. The results of the present study demonstrated that rosacea‐like dermatitis was rarely observed (less than 3%), although local side effects decreased over time in the switch areas. Even if poor medication adherence to topical therapy reduces treatment efficacy, the positive experience of using delgocitinib ointment may have contributed to improved medication adherence in the present study. The high ratings of use satisfaction by PPQ in this study (Figure 6) may also have influenced these improvements in treatment adherence. Moreover, skin condition was also maintained at a high level after the treatment. Taken together, these findings suggest that switching TCS to delgocitinib ointment was effective and led to higher treatment satisfaction for AD patients.

In the present study, a modest increase in the EASI, NRS, and ADCT scores was observed at 12 weeks compared with 8 weeks, which may be attributable to the inclusion of six patients who discontinued the study due to relapses or decreased medication adherence. Notably, all six patients had been using very strong TCS before switching. Therefore, when switching from very strong TCS, the patient's condition should be evaluated carefully, and a gradual switch or combination of TCS with other therapies may be required. Regarding side effects, delgocitinib ointment requires caution when used in patients with an infection, including local acne, folliculitis, herpes simplex, and Kaposi's varicelliform eruption. These side effects are generally expected to resolve without treatment [7, 8, 9, 10]. In this study, a side effect (folliculitis) was observed in one patient.

The low molecular weight of delgocitinib allows it to be easily absorbed through the skin; this has led to concerns about systemic effects, particularly the potential risk for malignant tumors, which has been noted with oral JAK inhibitors [21, 22]. However, a study of delgocitinib 0.5% ointment applied twice daily (up to 5 g per dose) in patients with AD reported the proportion of patients with detectable plasma concentrations of delgocitinib was approximately 12% at 4 weeks, 16% at 12 weeks, 14% at 28 weeks, and 11% at 52 weeks after treatment initiation. Among these patients, plasma concentrations remained near the lower limit of quantification, indicating that only a minimal amount of delgocitinib entered the bloodstream [8, 9]. During the clinical study, administration of doses exceeding 5 g, corresponding to treatment coverage of approximately 30% of the body surface area, was not investigated. For this reason, at the time of approval, delgocitinib was limited to twice daily applications, with a maximum dose of 5 g per application. This dosage limitation may be insufficient for treating extensive rashes in some cases. If a rash extends over a broad area during the acute phase, a potential treatment strategy may involve rapidly reducing inflammation with TCS or other medications, followed by early transition to delgocitinib ointment. Moreover, the efficacy and safety of combination therapy with delgocitinib ointment and other therapeutic drugs and the roles of delgocitinib ointment in proactive therapy need to be investigated further. Moreover, the present study has potential bias in patient selection. Screening and enrollment were conducted by physicians at each site, which may have introduced selection bias related to disease severity, treatment history, and medication adherence. In addition, as this was a single‐arm, open‐label study, the potential influence of placebo effects and observer biases cannot be ruled out.

In summary, the present study provides strong evidence supporting the efficacy of switching from TCS to delgocitinib ointment. Although our study had a limited sample size and observation period, larger clinical studies and long‐term observational studies are needed in the future. More importantly, it is essential to use TCS of appropriate potency, depending on the affected regions of the trunk and extremities. In contrast, delgocitinib ointment may not require such considerations, offering a simpler treatment strategy and potentially improving patient adherence. Additionally, delgocitinib ointment is well‐suited for cases requiring extensive application and for multiple applications of TCS depending on the location of the skin rash. Thus, we conclude that delgocitinib ointment is an effective treatment option for patients with AD presenting with rashes on the trunk and extremities, as well as for those concerned about the potential side effects of TCS.

## Ethics Statement

The protocol and ethical considerations of the study were reviewed and approved by a certified clinical research ethics committee (certification number: CRB3210001).

## Consent

The study was conducted in accordance with informed consent policies, which allow all participants who provide written documents with a signature prior to study enrollment to participate.

## Conflicts of Interest

M.A. has received research grants, consulting fees, speaker fees, and/or participated in clinical trials for Amgen, Maruho, AbbVie, Kyowa Kirin, TORII, LEO Pharma, Eli Lilly, Bristol Myers Squibb, Novartis, Sun Pharma, Sanofi, and UCB Pharma. A.I. has received advisory board honoraria, consulting fees, or speaker honoraria from AbbVie, Eli Lilly, Japan Tobacco, Maruho, Novartis, Sanofi, LEO Pharma, and TORII Pharmaceutical; he has also received research grants from AbbVie, Eli Lilly, Japan Tobacco, Novartis, Otsuka Pharmaceutical, Amgen, and Sanofi. K.K. has received consulting fees, honoraria, grant support, and/or lecture fees from AbbVie, Amgen, Eli Lilly, Kyowa Kirin, Japan Tobacco, LEO Pharma, Maruho, Mitsubishi Tanabe, Ono Pharmaceutical, Pfizer, Procter & Gamble, Sanofi, Regeneron, Taiho, and TORII Pharmaceutical. H.S. has received speaker's fees from AbbVie, Sanofi, Bristol‐Myers Squibb, Eli Lilly, Maruho, Nippon Boehringer Ingelheim, Taiho Pharmaceutical, Otsuka Pharmaceutical, Pfizer Japan, TORII Pharmaceutical, UCB Japan, Amgen, Mitsubishi Tanabe Pharma, LEO Pharma, and Novartis; research grants (clinical trials) from AbbVie and LEO Pharma; scholarships from Sun Pharma Japan, Maruho, Taiho Pharmaceutical, and TORII Pharmaceutical; and is an Editorial Board member of The Journal of Dermatology and a co‐author of this article. To minimize bias, he was excluded from all editorial decision making related to the acceptance of this article for publication. H.K. and H.T. are employees of TORII Pharmaceutical Co. Ltd. While Japan Tobacco Inc. is the parent company of TORII Pharmaceutical, and LEO Pharma holds the commercial right to delgocitinib cream (Anzupgo) outside Japan, neither Japan Tobacco Inc. nor LEO Pharma was involved in the conduct or interpretation of this study.

## Data Availability

The data underlying this article cannot be shared publicly to protect the privacy of individuals who participated in the study. The data will be shared upon reasonable request to the corresponding author.
